# Multi-objective optimization of dimensional accuracy and part weight in injection molding of a 3D curved shin guard plate

**DOI:** 10.1038/s41598-026-58677-7

**Published:** 2026-06-22

**Authors:** Son Hoang, Vinh Quang Pham, Cong Chi Tran

**Affiliations:** 1 Faculty of Electronics Engineering 1, Posts and Telecommunications Institute of Technology, Hanoi, Vietnam; 2 Institute of Optics, Mechanics and Specialized Materials, Department of Security Industry, Ministry of Public Security, Hanoi, Vietnam; 3 Faculty of Electromechanical and Civil Engineering, Vietnam National University of Forestry, Hanoi, Vietnam

**Keywords:** Injection molding, Multi-objective optimization, NSGA-II, Response surface methodology, Entropy-weighted TOPSIS, Engineering, Mathematics and computing

## Abstract

Injection molding of complex three-dimensional curved components is highly sensitive to processing conditions, particularly when dimensional accuracy and packing quality must be balanced simultaneously. This study proposes an integrated optimization and decision-making framework to improve the molding quality of a commercial curved shin guard plate (SGP) by combining Response Surface Methodology (RSM), adaptive non-dominated sorting genetic algorithm II (NSGA-II), and entropy-weighted Technique for Order Preference by Similarity to Ideal Solution (TOPSIS). A Box–Behnken design was employed to investigate the effects of packing pressure (*PP*), melt temperature (*MT*), and cooling time (*CT*) on absolute dimensional deviation (*ΔD*) and molded part weight (*PW*). The results showed that the developed quadratic response surface models exhibited good predictive capability, and analysis of variance identified *PP* as the most influential parameter. The surrogate models were integrated with standard and adaptive NSGA-II algorithms to perform multi-objective optimization and generate well-distributed Pareto-optimal solutions, with the adaptive NSGA-II showing improved Pareto-front exploration capability based on crowding-distance and hypervolume analyses. Entropy-weighted TOPSIS was subsequently applied to identify the most suitable compromise solution. Experimental validation was conducted at the TOPSIS-selected optimum together with two additional Pareto-optimal conditions. The validation results showed satisfactory agreement between predicted and experimental responses, with deviations remaining below 7.41% for *ΔD* and below 1.70% for *PW*. These results demonstrate the practical applicability of the proposed optimization framework for complex injection-molded components under industrial manufacturing conditions.

## Introduction

Injection molding remains one of the most widely used manufacturing processes for thermoplastic components requiring high productivity and accurate replication of complex geometries^1,2^. Its industrial applications have expanded beyond conventional automotive and consumer products toward personal protective equipment and anatomically contoured parts, where dimensional fidelity and geometric conformity directly influence product functionality and user comfort. However, the injection molding process is strongly governed by thermo-rheological and pressure-dependent phenomena. Variations in packing pressure, melt temperature, and cooling conditions may lead to non-uniform solidification, residual stresses, and post-ejection deformation, which are typically manifested as dimensional deviation, shrinkage, or warpage^3,4^.

Previous studies have extensively investigated the influence of processing parameters on deformation and dimensional stability in injection-molded components. For instance, Kurtaran and Erzurumlu^5^ combined Response Surface Methodology (RSM) with genetic algorithms to reduce warpage in thin-shell plastic parts. Other studies have shown that packing or holding pressure significantly affects density evolution and material distribution during the packing stage, thereby influencing the final mass and structural compactness of molded products^6,7^. Meanwhile, cooling conditions play a crucial role in governing heat dissipation, residual stress relaxation, and dimensional stabilization during solidification, which directly affects shrinkage and warpage behavior in molded parts^8^. Comprehensive reviews have also highlighted the increasing use of simulation-assisted and data-driven optimization techniques for improving dimensional quality and minimizing deformation in injection molding processes^4^.

To efficiently explore complex process parameter spaces, surrogate-assisted optimization frameworks have become increasingly popular. In such approaches, design-of-experiments (DOE) and response surface methodology are often used to construct predictive models that approximate the relationships between processing conditions and product quality. These models are subsequently integrated with metaheuristic algorithms to identify optimal parameter combinations while reducing computational cost. Recent studies have demonstrated that combining surrogate models with evolutionary optimization techniques can effectively improve product quality and process efficiency in injection molding processes^9,10^. Among various evolutionary algorithms, the non-dominated sorting genetic algorithm II (NSGA-II) has been widely adopted for solving multi-objective optimization problems in manufacturing systems due to its ability to simultaneously handle conflicting objectives and maintain solution diversity^11–13^. Several studies have successfully applied NSGA-II and related multi-objective optimization approaches to injection molding parameter optimization, enabling the identification of well-distributed Pareto-optimal solutions for improving dimensional accuracy and process performance^14–16^. Pareto-based approaches are particularly suitable when multiple quality indicators must be considered simultaneously, since improving one objective may degrade another.

Despite the considerable progress in optimization techniques for injection molding processes, several challenges remain when these frameworks are applied to complex three-dimensional curved components used in practical industrial environments. First, many studies evaluate deformation mainly through global warpage indicators obtained from numerical simulations. In industrial inspection practice, however, dimensional quality is often assessed using localized dimensional deviations measured at tolerance-critical locations^17^. A molded component may therefore exhibit acceptable global warpage while still failing dimensional inspection due to excessive deviation in specific curvature transitions or edge regions. Such tolerance-oriented evaluation criteria are rarely incorporated explicitly into optimization formulations^4,8^.

Second, most optimization studies primarily focus on geometric deformation metrics while paying comparatively less attention to molded part weight as an indicator of packing effectiveness and material densification. Under a constant cavity volume, variations in part mass reflect differences in melt compression and packing behavior during the post-filling stage. Adjustments in packing pressure or cooling time intended to reduce dimensional deviation may simultaneously alter the amount of material retained in the cavity, thereby influencing the final part weight and structural compactness^6,18^. Considering dimensional deviation together with part weight therefore provides a more comprehensive description of the interactions between process parameters and product quality^18^.

A third limitation concerns the transition from optimization results to practical decision-making. Algorithms based on Pareto dominance, such as NSGA-II, usually produce multiple non-dominated solutions that represent different compromises among the objectives. However, many studies conclude with the Pareto front without providing a systematic procedure for selecting a single operating condition suitable for industrial implementation^19^. In practical production environments, engineers require a transparent and reproducible decision-making strategy to translate the Pareto solution set into a final parameter combination^20,21^.

To address these limitations, this study investigates a commercial three-dimensional curved shin guard plate (SGP) and proposes an integrated optimization and decision-making framework for the injection molding process. Two practically relevant objectives are considered: absolute dimensional deviation (*ΔD*), evaluated at multiple inspection points, and molded part weight (*PW*), which reflects packing effectiveness and material densification under constant cavity volume. A Box–Behnken design is employed to explore the effects of packing pressure, melt temperature, and cooling time. Quadratic response surface models are then developed and statistically validated, followed by multi-objective optimization using NSGA-II to generate the Pareto solution set. An entropy-weighted Technique for Order Preference by Similarity to Ideal Solution (TOPSIS) method is subsequently applied to identify the most suitable compromise solution, and experimental validation is conducted to verify the reliability of the proposed framework.

Although hybrid frameworks combining RSM, NSGA-II, and TOPSIS have been widely reported in injection molding optimization studies, most previous works primarily focus on standard benchmark geometries or simulation-oriented optimization objectives^5,14–16^. For example, Kurtaran and Erzurumlu^5^ investigated warpage reduction of thin-shell components using RSM and genetic algorithms, while Li, et al.^15^ and Jung, et al.^16^ employed simulation-driven multi-objective optimization strategies for conventional molded parts. In contrast, the present study focuses on a commercial three-dimensional curved protective component under practical manufacturing conditions, where dimensional conformity is evaluated using experimentally measured tolerance-critical locations together with packing-related quality assessment. Another important limitation of previous studies is that Pareto-optimal solutions are often generated without a systematic and objective procedure for selecting a final operating condition suitable for industrial implementation^19,22^. Although several recent studies have incorporated TOPSIS or related multi-criteria decision-making (MCDM) approaches for ranking Pareto solutions, the weighting factors are often predefined or selected empirically, which may introduce subjective bias into the final decision process^23,24^. In the present study, entropy-weighted TOPSIS is employed to provide a transparent and reproducible post-Pareto decision-making strategy without requiring subjective weighting assignment. To further clarify the positioning and distinctive contributions of the present study relative to recent literature, a comparative summary of representative multi-objective injection molding optimization studies is provided in Table 1.

**Table 1:** Comparison of recent multi‑objective optimization studies with the proposed framework.

Study	Product geometry	Quality objective(s)	How quality is evaluated?	Surrogate model	Optimization algorithm	MCDM method	Physical validation
^9^	Thin-walled plastic part	Warpage	Simulation	RSM + ANN	MOGA (hybrid)	None	No
^14^	Electrical socket	Filling time, volumetric shrinkage	Simulation	RSM	NSGA-II	None	No
^15^	Transparent multi-cavity part	Warpage, volume shrinkage	Simulation	Kriging	NSGA-II	None	No
^16^	Fan blade	Warpage, cycle time, clamping force	Simulation	Bayesian	NSGA-III	None	No
^19^	Dashboard	Warpage, shrinkage	Simulation	RSM + ANN	PSO	None	No
^23^	Moderately thick plane lens	Warpage, volume shrinkage	Simulation	PSO-BPNN	OMOPSO	TOPSIS (equal weight)	No
^24^	GFRP injection molded part	Warpage, shrinkage	Simulation	GA-ELM	MOFA	GRA-TOPSIS	No
^25^	Industrial part	Cycle time, temperature differential	Real-time sensor data	RSM + Bayesian	NSGA-II/ADoE	None	Yes
^26^	Automotive battery box	Dimensional stability, warpage, elongation	Physical measurement	Random Forest	Parameter analysis	None	Yes
This study	Shin guard plate	ΔD, PW	Physical measurement	RSM	Standard & Adaptive NSGA-II	Entropy weighted TOPSIS	Yes

*ANN* artificial neural network, *MOGA* multi-objective genetic algorithm, *BPNN* back-propagation neural network, *OMOPSO* optimized multi-objective particle swarm optimization, *GRA* grey relational analysis, *ELM* extreme learning machine, *MOFA* multi-objective firefly algorithm, *ADoE* adaptive design of experiments, *GFRP* glass fiber reinforced plastic.

The main contributions of this study are as follows. First, a practical framework integrating experimental design, adaptive multi-objective optimization, and entropy-based decision-making is developed for complex curved injection-molded components. Second, dimensional quality is assessed using tolerance-critical local deviations measured on physical parts rather than solely relying on simulation-based global warpage indicators. Third, molded part weight is incorporated as an additional objective to better represent packing effectiveness and material densification. Fourth, quantitative diversity analysis based on crowding-distance and hypervolume metrics is performed to compare the exploration capability of Standard and Adaptive NSGA-II algorithms. Finally, the proposed framework is experimentally validated on a commercial protective component, demonstrating its applicability to real manufacturing conditions.

## Methods

### Product description and injection molding setup

**Fig. 1:**
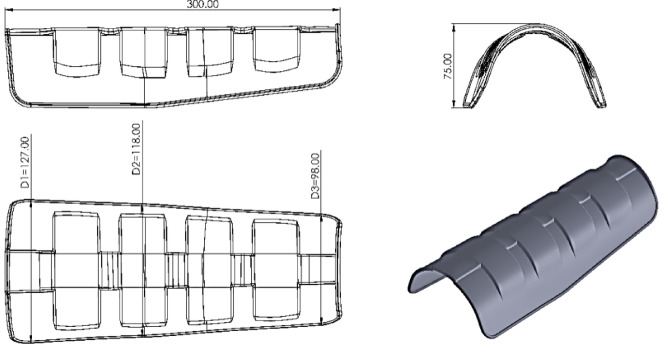
CAD model of the SGP and locations of dimensional measurements (*D1*,* D2*, and *D3*).

The component investigated in this study is a SGP product, a rigid plastic protective part designed to cover the anterior region of the lower leg. In practical use, the part is required to conform closely to the wearer’s leg geometry, making dimensional accuracy a critical performance requirement. The three-dimensional computer-aided design (CAD) model of SGP is shown in Fig. 1.

The SGP features a complex three-dimensional curved geometry with a non-uniform thickness distribution. The primary curvature follows the longitudinal direction of the leg, while additional curvature variations occur across the width of the part. Thinner sections are mainly located in the central region, whereas thicker zones appear near the edges and locally reinforced areas. Such geometric characteristics promote non-uniform melt flow and cooling behavior during injection molding. Thin regions tend to solidify earlier, while thicker sections remain molten for a longer period, which can result in uneven shrinkage and deviations from the nominal geometry after demolding.

The material used in this study was polypropylene, a semi-crystalline thermoplastic known for its favorable stiffness-to-weight ratio, impact resistance, and processability. The base polypropylene resin was dry-blended with a black masterbatch (LP9030) at approximately 0.6 wt% prior to molding to obtain uniform black coloration. The blended material was also dried at 80 °C to remove residual moisture, ensuring stable melt behavior, improved surface finish, and dimensional consistency.

**Fig. 2:**
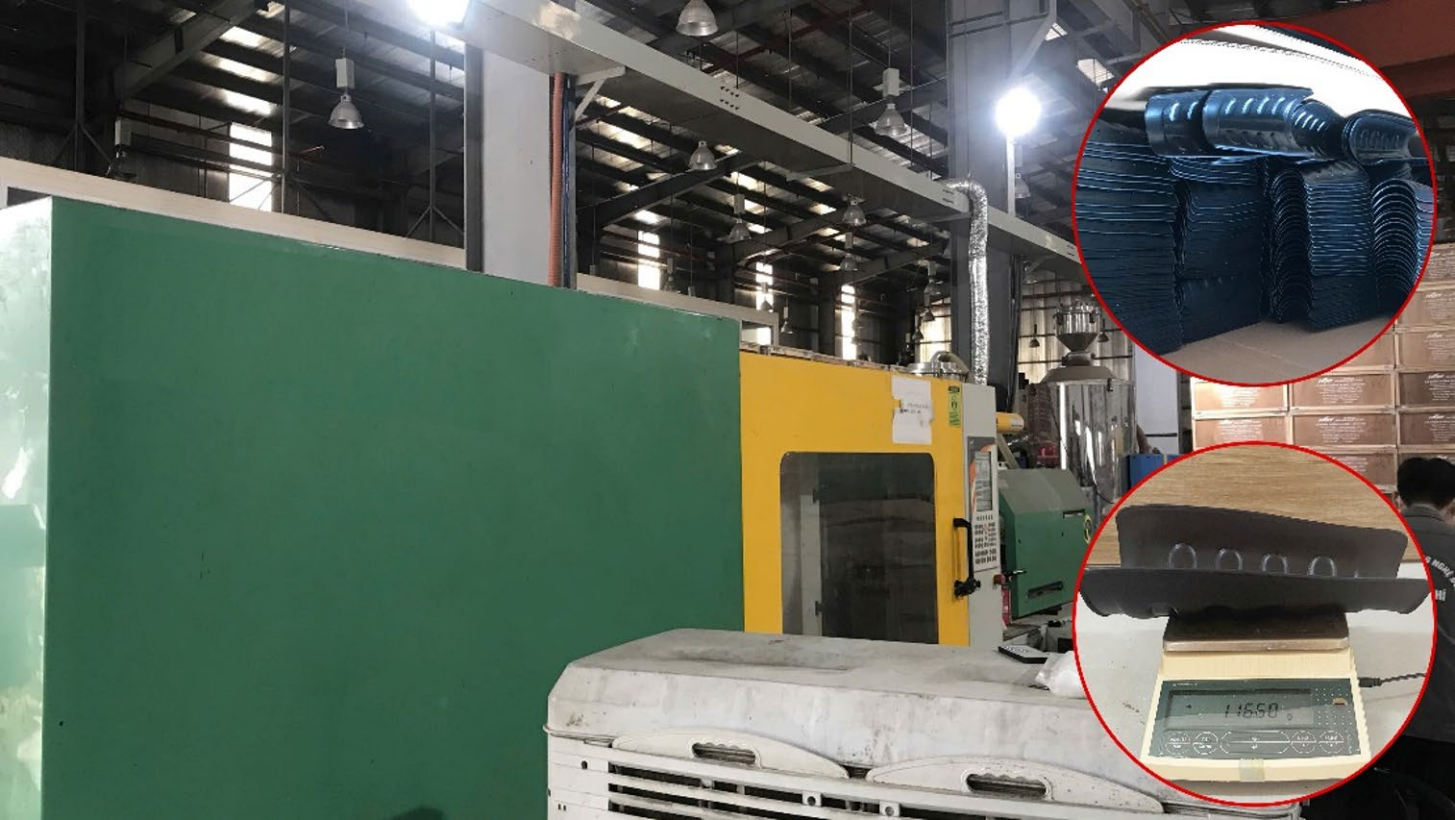
Injection molding machine and molded SGP used for experimental validation.

To quantitatively evaluate these effects, *ΔD* was selected as a primary quality indicator to describe local deviations between the molded part and the nominal design at predefined measurement locations. In addition to *ΔD*,* PW* was adopted as an indirect indicator of packing effectiveness and material densification during the post-filling stage. Under a fixed cavity volume and stable molding conditions, variations in part weight primarily reflect differences in melt compression, density evolution, and material retention before gate freeze-off. Insufficient packing typically leads to increased shrinkage and reduced final mass, whereas adequate packing compensates for volumetric contraction and results in higher part weight.

The injection molding experiments were conducted using a conventional reciprocating screw injection molding machine equipped with a single-cavity mold specifically designed for the SGP (Fig. 2). The mold was fabricated from tool steel and integrated with internal cooling channels following standard industrial practice. Prior to experimentation, the machine and mold were stabilized to ensure steady processing conditions. The polymer pellets were pre-dried following the material supplier’s guidelines to reduce the influence of residual moisture during processing.

### Experimental design

A three-factor Box–Behnken design (BBD) was employed to investigate the influence of key processing parameters on injection molding performance while minimizing the number of experimental runs. The BBD method enables efficient estimation of quadratic response surfaces and interaction effects, making it particularly suitable for multi-objective optimization of thermoplastic molding processes.

Based on preliminary trials, industrial practice, and literature recommendations, packing pressure (*PP*), melt temperature (*MT*), and cooling time (*CT*) were identified as the primary process variables. These parameters directly govern melt densification, flow behavior, and solidification, which are the dominant mechanisms affecting dimensional deviation and part weight in injection molding. Each factor was examined at three coded levels (− 1, 0, + 1), as summarized in Table 2. The selected ranges were determined according to machine capability and material processing guidelines to ensure stable molding conditions without flash formation, short shots, or excessive residual stresses.

**Table 2:** Factors and levels (BBD).

Factor	Unit	Coded levels		
		−1	0	+ 1
Packing pressure (*PP*)	bar	25	35	45
Melt temperature (*MT*)	°C	200	220	240
Cooling time (*CT*)	s	30	45	60

To isolate the effects of these variables, all other process parameters were maintained constant throughout the experimental campaign. Injection speed was kept constant to ensure comparable melt front advancement during cavity filling. Holding time was fixed at 5.0 s so that packing pressure remained the dominant densification parameter during the post-filling stage. Back pressure during plasticization was maintained at 8 bar to ensure homogeneous melt mixing, and the screw rotation speed was kept constant to maintain stable melting conditions. In addition, the clamping force was set to 500 tons to ensure cavity sealing stability and prevent flash formation.

In this study, *ΔD* and *PW* were selected as the primary quality indicators because they are directly associated with dimensional conformity, packing effectiveness, and material utilization in injection molding production. For the curved shin SGP, functional performance depends mainly on dimensional accuracy at tolerance-critical regions rather than on a single global warpage value. Therefore, *ΔD* measured at the selected inspection points was used to evaluate geometric conformity, while *PW* served as an indirect indicator of melt densification and shrinkage behavior. Although additional quality indicators such as residual stress and full-field warpage are also important, the combined use of *ΔD* and *PW* was considered sufficient to establish a practical and experimentally measurable optimization framework for industrial manufacturing conditions.

*ΔD* was used to quantify the geometric deviation between the molded part and the nominal CAD model. Due to the complex three-dimensional geometry of the SGP, dimensional deviation was evaluated at discrete representative locations rather than across the entire surface. The three inspection points (*D1*, *D2*, and *D3*) were selected as tolerance-critical locations based on the product geometry and practical quality-control requirements specified by the manufacturer, representing the edge, intermediate, and central regions of the curved SGP, respectively (Fig. 1).

At each location * i*, the local dimensional deviation $\:{\Delta\:}{D}_{i}$ was defined as the absolute difference between the measured dimension and the nominal CAD value:

1 $$\:{\Delta\:}{D}_{i}=\mid\:{D}_{i,M}-{D}_{i,CAD}\mid\:$$

where $\:{D}_{i,\mathrm{M}}$ represents the measured dimension of the molded part and $\:{D}_{i,\mathrm{CAD}}$ denotes the nominal dimension obtained from the CAD model.

The overall dimensional deviation $\:{\Delta\:}D\:$for each molded part was calculated as the arithmetic mean of the local deviations across all measurement locations:

2 $$\:{\Delta\:}D=\frac{1}{n}\sum_{i=1}^{n}{\Delta\:}{D}_{i}$$

where $\:n\:$represents the number of measurement locations.

For each experimental condition, three parts were produced, and the *ΔD* value of each part was measured. The mean and standard deviation (*SD*) were then calculated from the three replicates.

In addition to dimensional accuracy, *PW* was adopted as a second response variable to characterize the packing effectiveness and material densification during the molding process. Under constant cavity volume, variations in molded part mass primarily reflect differences in material distribution and density evolution during the packing and cooling stages. Consequently, part weight provides a practical indicator of how processing conditions influence the compactness and material retention within the mold cavity. The mass of each part was measured using a precision electronic balance with a resolution of 0.01 g. For each experimental condition, three parts were produced and weighed, and the average value was used as the representative part weight for that run, as expressed by:

3 $$\:PW=\frac{1}{k}\sum_{j=1}^{k}{m}_{j}$$

where $\:{m}_{j}$ is the measured mass of specimen *j* and *k* is the number of replicated samples. The *SD* of the three replicates was also calculated for * PW* to quantify experimental uncertainty.

To ensure measurement repeatability and to reduce uncertainty, all molded specimens were conditioned at room temperature (25 °C) for 24 h prior to dimensional measurement and mass determination. The measured values were then employed as response variables in the regression analysis.

### Response surface modeling

RSM was performed to model the relationship between the injection molding parameters and the responses. Based on the Box–Behnken experimental data, quadratic regression models were fitted to describe the effects of the process variables on *ΔD* and *PW*. The general quadratic model is defined as:

4 $$\:Y={\beta\:}_{0}+\sum_{i=1}^{k}{\beta\:}_{i}{X}_{i}+\sum_{i=1}^{k}{\beta\:}_{ii}{X}_{i}^{2}+\sum_{i<j}^{k}{\beta\:}_{ij}{X}_{i}{X}_{j}$$

where * Y* represents the predicted *ΔD* and *PW*, $\:{\beta\:}_{0}$is the intercept term, $\:{\beta\:}_{i}$, $\:{\beta\:}_{ii}$, and $\:{\beta\:}_{ij}$ denote the linear, quadratic, and interaction regression coefficients, respectively; $\:{X}_{i}$ and $\:{X}_{j}$ are the values of *PP*,* MT*, and *CT.*

The regression coefficients of the response surface models were estimated using the least-squares method. Statistical analysis of variance (ANOVA) was then performed to evaluate the significance of the models and the contribution of individual terms. The statistical significance of each factor was assessed using the p-value at a confidence level of 95%. The developed response surface models were subsequently used as surrogate functions in the multi-objective optimization stage, enabling efficient exploration of the design space without requiring additional computationally expensive injection molding simulations.

### Multi-objective optimization using NSGA-II and adaptive NSGA-II

In this study, the NSGA-II was employed to solve the formulated multi-objective optimization problem. The optimization aimed to simultaneously minimize *ΔD* and maximize *PW* of the injection-molded protective component.

The optimization was performed using the surrogate response surface models established from the DOE results, which describe the relationships between the processing parameters and the quality responses. Three decision variables were considered in the optimization process, namely *PP*,* MT*, and *CT*. Their lower and upper bounds were defined as 25–45 bar for *PP*, 200–240 °C for *MT*, and 30–60 s for *CT*.

The multi-objective optimization problem can therefore be formulated as:

5 $$\begin{aligned} \min f_{1} = & \Delta \:D(PP,MT,CT) \\ \:\max f_{2} = & PW(PP,MT,CT) \\ \end{aligned}$$

In injection molding with a fixed cavity geometry, part weight is closely related to the packing condition and volumetric shrinkage of the polymer melt. Insufficient packing pressure or premature solidification typically leads to incomplete cavity filling and increased shrinkage, resulting in a reduction in the final part weight. Therefore, maximizing *PW* can be considered an indirect indicator of improved packing quality and reduced warpage-related deformation.

NSGA-II evolves candidate solutions through selection, crossover, and mutation. During each generation, solutions are classified using non-dominated sorting, and diversity along the Pareto front is maintained by the crowding distance mechanism. Let $\:{P}_{t}$ denote the parent population at generation * t*. Offspring solutions $\:{Q}_{t}$ are generated through genetic operators. The two populations are then merged as $\:{R}_{t}={P}_{t}\cup\:{Q}_{t}$ and sorted according to non-dominated fronts $\:{F}_{1},{F}_{2},\dots\:,{F}_{k}$. The next generation population $\:{P}_{t+1}$ is constructed by sequentially selecting solutions from these fronts until the predefined population size is reached. Within each front, solutions with larger crowding distances are preferred in order to maintain diversity along the Pareto front^25^.

To ensure a fair comparison between algorithm variants, the random number generator was initialized using a fixed seed. Two NSGA-II variants were implemented: the standard NSGA-II and an adaptive NSGA-II with a dynamic mutation strategy. In both cases, the population size was set to 200 individuals and the maximum number of generations was 500. Tournament selection with a size of two was used for parent selection. Simulated Binary Crossover (SBX) was applied with a crossover probability of 0.9, while the distribution indices for crossover and mutation were both set to 10.

For the standard NSGA-II, polynomial mutation was performed with a fixed mutation probability of 0.1. In contrast, the adaptive NSGA-II dynamically adjusts the mutation probability during the evolutionary process to balance exploration and exploitation. The mutation probability gradually decreases as the generation progresses according to the following equation:

6 $$\:p_{m} \left( g \right) = p_{{\min }} + (p_{{\max }} - p_{{\min }} )\left( {1 - \frac{g}{{G_{{\max }} }}} \right)$$

where $\:{p}_{m}\left(g\right)$ is the mutation probability at generation * g*, $\:{p}_{max}$ and $\:{p}_{min}$ denote the maximum and minimum mutation probabilities, respectively, and $\:{G}_{max}$ represents the maximum number of generations.

In this study, the mutation probability decreases from $\:{p}_{max}=0.20\:$at the initial stage to $\:{p}_{min}=0.02\:$at the final stage. This adaptive mechanism increases population diversity during the early search stage and improves convergence stability in the later generations.

Using these settings, both algorithms generated a set of Pareto-optimal solutions representing the trade-off between dimensional accuracy and part weight. These Pareto solutions were subsequently analyzed using a multi-criteria decision-making method to determine the most balanced processing condition.

### Post-Pareto decision-making using entropy-weighted TOPSIS

Although the NSGA-II optimization generates a set of Pareto-optimal solutions representing the trade-off between *ΔD* and *PW*, practical manufacturing applications require the selection of a single compromise solution. To identify the most balanced operating condition, a multi-criteria decision-making (MCDM) method based on entropy-weighted TOPSIS was adopted.

The TOPSIS method evaluates candidate solutions based on their relative distances to the positive and negative ideal solutions. The positive ideal solution corresponds to the most desirable values of all objectives, whereas the negative ideal solution represents the least favorable performance across the objectives^26^.

Let $\:X=\left[{x}_{ij}\right]$denote the decision matrix obtained from the Pareto-optimal solution set, where $\:i=\mathrm{1,2},...,m\:$represents the candidate solutions and $\:j=\mathrm{1,2},...,n$ represents the evaluation criteria.

The decision matrix was first normalized using min–max normalization after converting cost and benefit criteria into benefit-type preference values:

7 $$\:{r}_{ij}=\left\{\begin{array}{cc}\frac{{x}_{ij}-{x}_{j}^{\mathrm{m}\mathrm{i}\mathrm{n}}}{{x}_{j}^{\mathrm{m}\mathrm{a}\mathrm{x}}-{x}_{j}^{\mathrm{m}\mathrm{i}\mathrm{n}}},&\:\mathrm{for\:benefit\:criteria}\\\:\frac{{x}_{j}^{\mathrm{m}\mathrm{a}\mathrm{x}}-{x}_{ij}}{{x}_{j}^{\mathrm{m}\mathrm{a}\mathrm{x}}-{x}_{j}^{\mathrm{m}\mathrm{i}\mathrm{n}}},&\:\mathrm{for\:cost\:criteria}\end{array}\right.$$

To objectively determine the relative importance of each criterion, entropy weighting was employed. The entropy value for each criterion is calculated as:

8 $$E_{j} = - k\mathop \sum \limits_{{i = 1}}^{m} p_{{ij}} \ln \left( {p_{{ij}} } \right)$$

Where $$p_{{ij}} = \frac{{r_{{ij}} }}{{\mathop \sum \nolimits_{{i = 1}}^{m} r_{{ij}} }}$$ and $$\:k=\frac{1}{\mathrm{l}\mathrm{n}\left(m\right)}$$ 

The corresponding entropy weight for each criterion is then determined as:

9 $$\:{w}_{j}=\frac{1-{E}_{j}}{\sum\:_{j=1}^{n}(1-{E}_{j})}$$

Using these weights, the weighted normalized decision matrix is obtained:

10 $$\:{v}_{ij}={w}_{j}{r}_{ij}$$

The positive ideal solution $\:{A}^{+}$and negative ideal solution $\:{A}^{-}$are then defined as:

11 $$\begin{gathered} \:A^{ + } = \{ \max (v_{{ij}} ){\mid }\:j \in \:J_{{benefit}} ,\min (v_{{ij}} ){\mid }\:j \in \:J_{{\cos t}} \} \hfill \\ \:A^{ - } = \{ \min (v_{{ij}} ){\mid }\:j \in \:J_{{benefit}} ,\max (v_{{ij}} ){\mid }\:j \in \:J_{{\cos t}} \} \hfill \\ \hfill \\ \end{gathered}$$

The Euclidean distances of each alternative from the ideal and negative ideal solutions are computed as:

12 $$\:{S}_{i}^{+}=\sqrt{\sum_{j=1}^{n}({v}_{ij}-{v}_{j}^{+}{)}^{2}}\:\:\:\:\:\:\:\:\:\:{S}_{i}^{-}=\sqrt{\sum_{j=1}^{n}({v}_{ij}-{v}_{j}^{-}{)}^{2}}$$

Finally, the relative closeness coefficient for each solution is calculated as:

13 $$\:{C}_{i}=\frac{{S}_{i}^{-}}{{S}_{i}^{+}+{S}_{i}^{-}}$$

where $\:{C}_{i}\:$denotes the TOPSIS closeness coefficient and $\:0\le\:{C}_{i}\le\:1$.

The Pareto-optimal solutions obtained from the NSGA-II optimization were evaluated using the entropy-weighted TOPSIS procedure, and the solution with the highest closeness coefficient was selected as the optimal processing parameter combination for the injection molding process.

## Results and discussion

### Statistical analysis and model development

The experimental results for *ΔD* and *PW* obtained from the 15 Box–Behnken design runs are summarized in Table 3. Each value is reported as the mean of three replicated measurements together with the corresponding *SD*. The *SD* values for *ΔD* ranged from 0.06 mm to 0.50 mm, while those for *PW* ranged from 0.55 g to 1.30 g, indicating acceptable experimental repeatability within the investigated processing window. These experimental data were subsequently used to develop the quadratic response surface models.

**Table 3:** Experimental results of *ΔD* and *PW* for each run (mean ± *SD*, *n* = 3).

Run	PP (bar)	MT (°C)	CT (s)	ΔD (mm)	PW (g)
1	35	200	30	1.03 ± 0.34	117.41 ± 1.24
2	45	220	30	0.49 ± 0.17	118.32 ± 0.84
3	35	220	45	0.62 ± 0.13	118.08 ± 0.88
4	45	240	45	0.35 ± 0.16	118.69 ± 0.57
5	25	220	30	1.65 ± 0.18	116.56 ± 1.06
6	35	220	45	0.64 ± 0.10	118.09 ± 0.84
7	25	200	45	1.32 ± 0.50	117.12 ± 0.58
8	35	240	60	0.55 ± 0.22	118.69 ± 0.98
9	35	220	45	0.63 ± 0.18	118.05 ± 1.11
10	45	200	45	0.57 ± 0.06	118.45 ± 0.57
11	45	220	60	0.53 ± 0.10	119.11 ± 0.93
12	25	240	45	1.11 ± 0.39	116.75 ± 1.30
13	35	240	30	0.64 ± 0.11	117.46 ± 1.07
14	35	200	60	0.66 ± 0.25	118.65 ± 0.55
15	25	220	60	0.86 ± 0.14	118.15 ± 1.15

Regression analysis based on RSM was performed to establish predictive relationships between the selected injection molding parameters and the two quality responses, namely *ΔD* and *PW*. The ANOVA results for *ΔD* are presented in Table 4. The regression model is statistically significant (*p* < 0.05). Among the investigated parameters, PP has the most significant influence on *ΔD*, followed by *MT* and *CT*. The model exhibits high goodness of fit with $\:{R}^{2}=0.9935$, $\:Adj\mathrm{-}{R}^{2}=0.9819$, and $\:Pred\mathrm{-}{R}^{2}=0.8979$. The coefficient of variation (*CV* = 2.39%) indicates satisfactory experimental precision, while the adequate precision value of 28.92 confirms a strong signal-to-noise ratio.

**Table 4:** ANOVA for the response surface model of *ΔD*.

Source	Sum of Squares	df	Mean Square	F-value	*p*-value
Model	1.74	9	0.1939	85.22	< 0.0001
*PP*	0.755	1	0.755	331.87	< 0.0001
*MT*	0.0222	1	0.0222	9.76	0.0261
*CT*	0.0721	1	0.0721	31.68	0.0025
*PP×MT*	0	1	0	0.011	0.9206
*PP×CT*	0.1722	1	0.1722	75.7	0.0003
*MT×CT*	0.0196	1	0.0196	8.62	0.0324
*PP²*	0.1264	1	0.1264	55.55	0.0007
*MT²*	0.0019	1	0.0019	0.8216	0.4063
*CT²*	0.0168	1	0.0168	7.39	0.0418
Residual	0.0114	5	0.0023		
Cor Total	1.76	14			

The regression equation describing *ΔD* as a function of the process variables is given in Eq. (14).

14 $$\begin{aligned} \Delta D = & 13.6638 - 0.2265PP - 0.040625MT \\ & - 0.136833CT - 1.25 \times \:10^{{ - 5}} PP \times \:MT \\ & + 0.00138333PP \times \:CT + 0.000233333MT \\ & \times \:CT + 0.00185PP^{2} + 5.625 \times \:10^{{ - 5}} MT^{2} \\ & + 0.0003CT^{2} \\ \end{aligned}$$

The ANOVA results for *PW* are summarized in Table 5. The regression model is statistically significant (*p* < 0.05). Among the investigated parameters, *PP* has the most dominant effect on part weight, since higher packing pressure improves cavity filling and compensates for polymer shrinkage during solidification. *MT* and *CT* also influence *PW* by affecting melt flow behavior and solidification conditions.

The developed model shows predictive capability with high $\:{R}^{2}=0.9943$, $\:Adj\mathrm{-}{R}^{2}=0.9840$, and $\:Pred\mathrm{-}{R}^{2}=0.9099$. The coefficient of variation is extremely low (*CV* = 0.0811%), indicating high experimental consistency. In addition, the adequate precision value of 34.70 confirms that the model has a strong signal and can be reliably used to navigate the design space.

**Table 5:** ANOVA for the response surface model of PW.

Source	Sum of Squares	df	Mean Square	F-value	*p*-value
Model	7.94	9	0.8827	96.48	< 0.0001
*PP*	3.63	1	3.63	396.79	< 0.0001
*MT*	0.062	1	0.062	6.78	0.048
*CT*	1.95	1	1.95	213.39	< 0.0001
*PP×MT*	0.093	1	0.093	10.17	0.0243
*PP×CT*	0.16	1	0.16	17.49	0.0086
*MT×CT*	0	1	0	0.0027	0.9603
*PP²*	0.1057	1	0.1057	11.55	0.0193
*MT²*	0.0849	1	0.0849	9.28	0.0285
*CT²*	0.0632	1	0.0632	6.91	0.0466
Residual	0.0457	5	0.0091		
Cor Total	7.99	14			

The predictive equation for *PW* is presented in Eq. (15).

15 $$\begin{aligned} \:PW = & 98.1313 + 0.0855417PP + 0.140271MT \\ & + 0.0365833CT + 0.0007625PP \times \:MT \\ & - 0.00133333PP \times \:CT - 8.33333 \times \:10^{{ - 6}} MT \\ & \times \:CT - 0.00169167PP^{2} - 0.000379167MT^{2} \\ & + 0.000581481CT^{2} \\ \end{aligned}$$

To further justify the selection of the surrogate model, additional statistical performance indicators were evaluated and are summarized in Table 6. Linear, two-factor interaction (2FI), and quadratic models were compared using predicted coefficient of determination (*R²)*, root mean squared error *(RMSE)*, mean absolute error *(MAE)*, mean absolute percentage error (*MAPE)*, corrected Akaike information criterion *(AICc)*, and Bayesian information criterion *(BIC)*. Among these, the quadratic model provided the highest predicted *R*² values together with the lowest prediction errors for both *ΔD* and *PW* responses, indicating improved predictive capability within the investigated processing range.

**Table 6:** Comparative statistical performance of Linear, 2FI, and Quadratic surrogate models.

Model	Response	*R*²	Adjusted *R*²	Predicted *R*²	RMSE	MAE	MAPE (%)	AICc	BIC
Linear	ΔD	0.8063	0.7535	0.6218	0.1508	0.1209	17.2193	−2.23	−3.39
	PW	0.9294	0.9101	0.8527	0.1957	0.1560	0.1323	5.36	4.19
2FI	ΔD	0.9155	0.8522	0.7041	0.0990	0.0882	13.2018	3.33	−7.72
	PW	0.9611	0.9319	0.8092	0.1446	0.1200	0.1020	14.43	3.39
Quadratic	ΔD	0.9935	0.9819	0.8979	0.0275	0.0231	3.3970	9.800	−38.12
	PW	0.9943	0.9840	0.9099	0.0566	0.0487	0.0413	30.68	−17.24

Although advanced surrogate models such as Kriging, Gaussian process regression (GPR), and ANN can capture highly nonlinear behaviors, these approaches generally require substantially larger datasets to ensure stable training and avoid overfitting. In the present study, the Box–Behnken design generated only 15 experimental runs due to the high cost and time associated with injection molding experiments. Therefore, the quadratic RSM model was considered a practical and statistically appropriate choice for local response approximation within the investigated processing parameters.

**Fig. 3:**
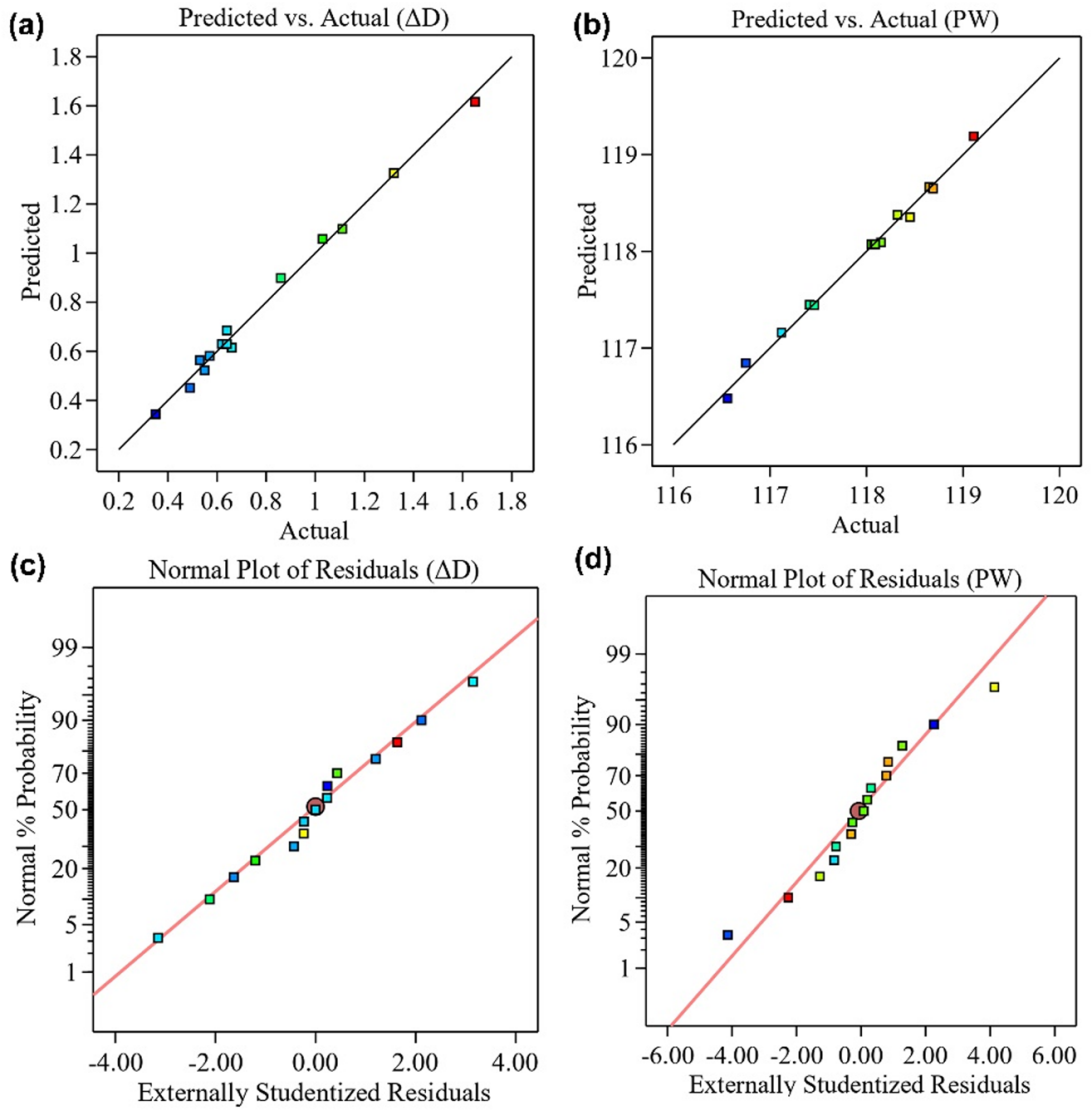
Predicted versus actual values for *ΔD* (**a**) and *PW* (**b**) together with normal probability plots of residuals for *ΔD* (**c**) and *PW* (**d**).

The validity of the developed regression models was further evaluated using the diagnostic plots presented in Fig. 3. The predicted versus experimental values of *ΔD* and PW are shown in Fig. 3a and b, respectively. In both cases, the data points are closely distributed around the 45° reference line, indicating satisfactory predictive capability of the developed models. In addition, the normal probability plots of residuals presented in Fig. 3c and d exhibit an approximately linear distribution, confirming that the residuals follow a normal distribution and that the underlying regression assumptions are reasonably satisfied.

Based on these statistical analyses, the response surface models can be used as surrogate functions for the subsequent multi-objective optimization using NSGA-II and adaptive NSGA-II.

### Effect of process parameters on *ΔD* and *PW*

**Fig. 4:**
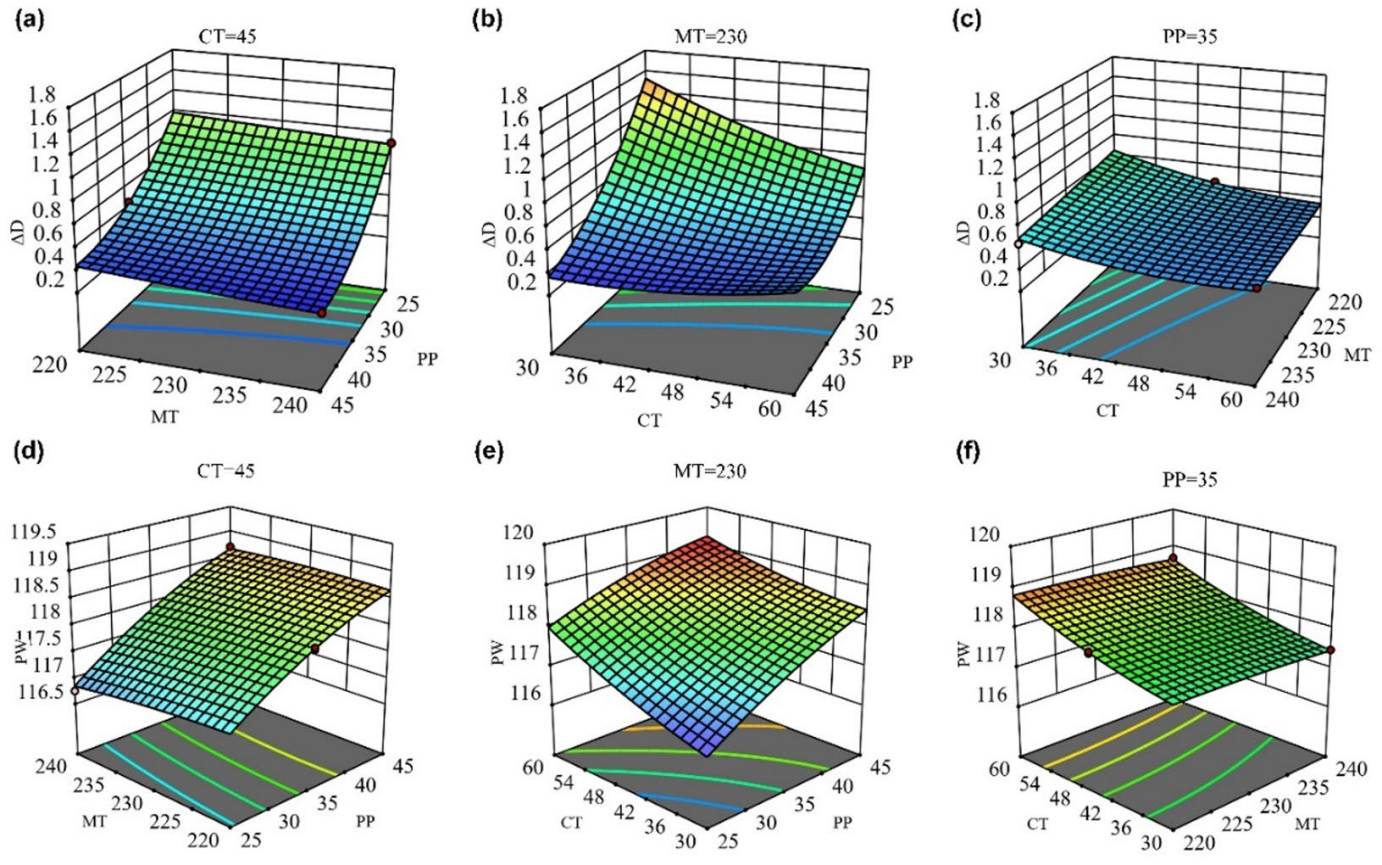
Effects of process parameters on *ΔD* (**a**, **b**, **c**) and *PW* (**d**, **e**, **f**).

The combined effects of the process parameters on *ΔD* and *PW* are shown in Fig. 4. These plots illustrate how variations in the main processing parameters influence both dimensional accuracy and material retention during mold filling and solidification. Among the investigated parameters, *PP* exhibits the most significant influence on both *ΔD* and *PW*. Increasing *PP* consistently reduces *ΔD* while simultaneously increasing *PW* across the investigated parameter range. This behavior is primarily associated with improved melt densification during the packing stage. A higher packing pressure enhances pressure transmission through the molten polymer, forcing additional melt into the cavity before gate freeze-off. As a semi-crystalline thermoplastic, *PP* also undergoes volumetric contraction during cooling due to crystallization. Higher packing pressure reduces the specific volume of the molten polymer and enhances shrinkage compensation before solidification, thereby improving dimensional stability. As a result, the final part mass increases and dimensional deviation after demolding is reduced^4,6,18^. The response surfaces further indicate that improved material densification under higher packing conditions directly contributes to enhanced dimensional accuracy.

*CT* also plays an important role, particularly through its interaction with PP. A longer cooling time allows more uniform heat dissipation within the molded part and reduces temperature gradients during solidification. This promotes more uniform shrinkage and enables partial relaxation of residual stresses before part ejection. Residual stresses in injection-molded parts primarily originate from non-uniform cooling and flow-induced molecular orientation during cavity filling^27^. Longer cooling time allows more complete solidification and stress relaxation prior to demolding, thereby reducing frozen-in stresses and improving dimensional stability. Consequently, higher *PW* values and improved dimensional stability are observed at extended cooling durations, especially under elevated *PP* levels. This interaction behavior is consistent with previous studies reporting that adequate cooling conditions help stabilize the internal stress distribution and reduce post-ejection deformation^4,28^.

In contrast, *MT* exhibits a comparatively weaker effect on both responses within the investigated range. Increasing *MT* reduces melt viscosity and improves flowability during cavity filling. For semi-crystalline polypropylene, melt temperature also influences crystallization behavior and molecular mobility during cooling. Higher melt temperatures improve cavity filling uniformity; however, excessive thermal energy may prolong solidification and increase thermal shrinkage variation, which can contribute to dimensional instability and residual stress development^29,30^. However, once the cavity is sufficiently filled, the influence of *MT* becomes less dominant than that of *PP* and *CT*, which primarily govern melt compression and shrinkage compensation during the packing and cooling stages^31^. This observation suggests that, for the present curved geometry, the final dimensional accuracy and material retention are mainly controlled by packing efficiency and cooling-driven solidification rather than by melt temperature variations.

However, in practical manufacturing environments, minimizing *ΔD* and maximizing *PW* must also be balanced against production efficiency and operating cost. In particular, *CT* directly influences the overall molding cycle. Although extending *CT* improves dimensional stability and residual stress relaxation, it simultaneously increases manufacturing time per part. Therefore, the trade-off between product quality and production efficiency becomes an important industrial consideration. The multi-objective optimization framework presented in the following section addresses this issue by generating a set of Pareto-optimal solutions, allowing manufacturers to select processing conditions according to specific production priorities, such as quality-oriented or high-throughput manufacturing.

### Multi-objective optimization using NSGA-II

**Fig. 5:**
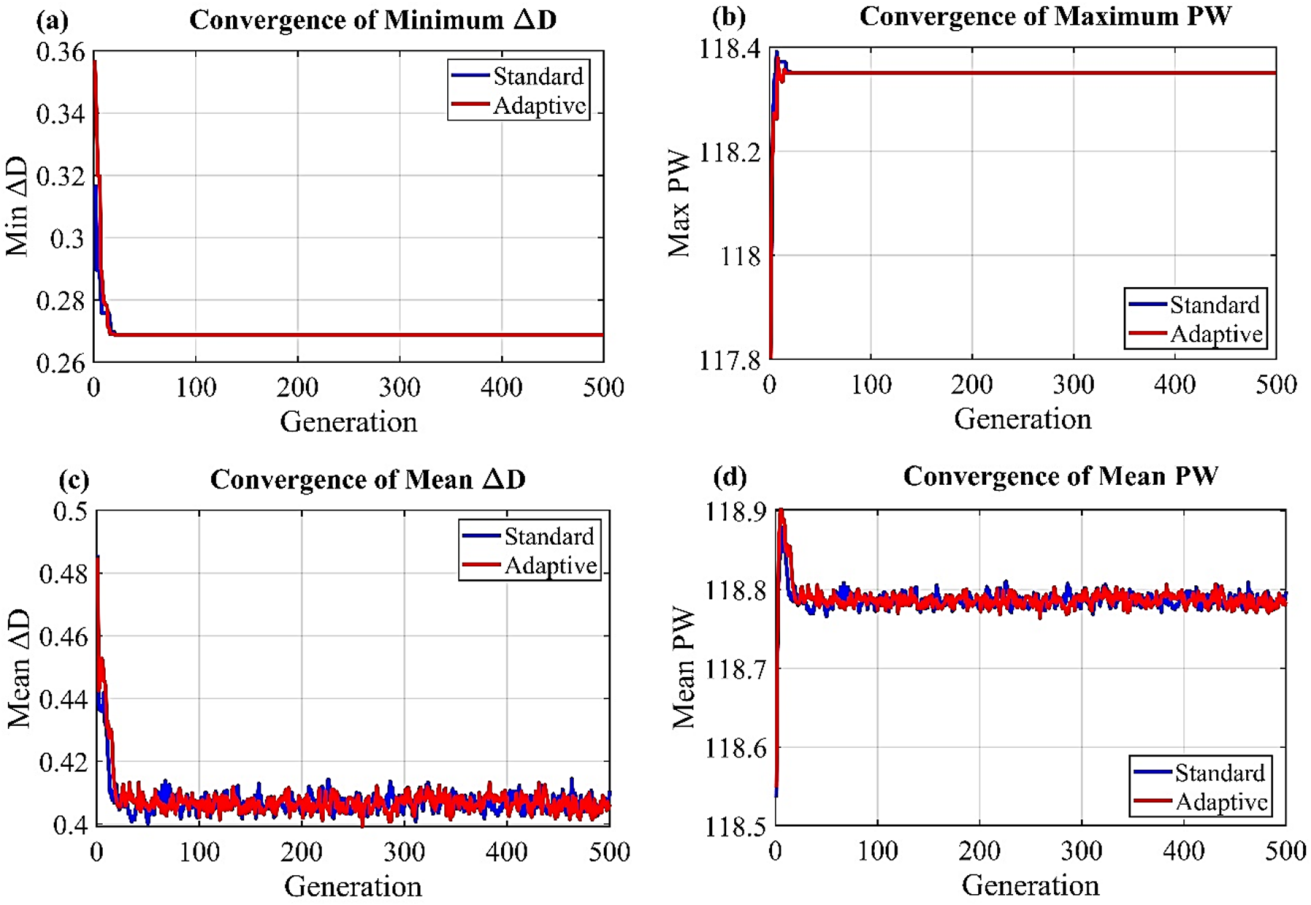
Convergence behavior of standard and adaptive NSGA-II algorithms for *ΔD* and *PW*.

The performance of the standard and adaptive NSGA-II algorithms was compared with respect to convergence, Pareto front distribution, and solution diversity. The corresponding convergence trends for both algorithms are illustrated in Fig. 5. The results indicate that the minimum *ΔD* decreases rapidly during the early generations and stabilizes after approximately 30–40 generations. A similar trend is observed for *PW*, where the maximum part weight quickly reaches a stable level and remains nearly constant thereafter. The evolution of the population mean values for both objectives further confirms that the search process converges toward a stable region with only minor fluctuations. These observations demonstrate that both algorithms efficiently approach the Pareto-optimal region within a relatively small number of generations. The adaptive mutation strategy exhibits slightly faster stabilization during the early search stage, which can be attributed to the higher mutation probability that enhances exploration capability.

**Fig. 6:**
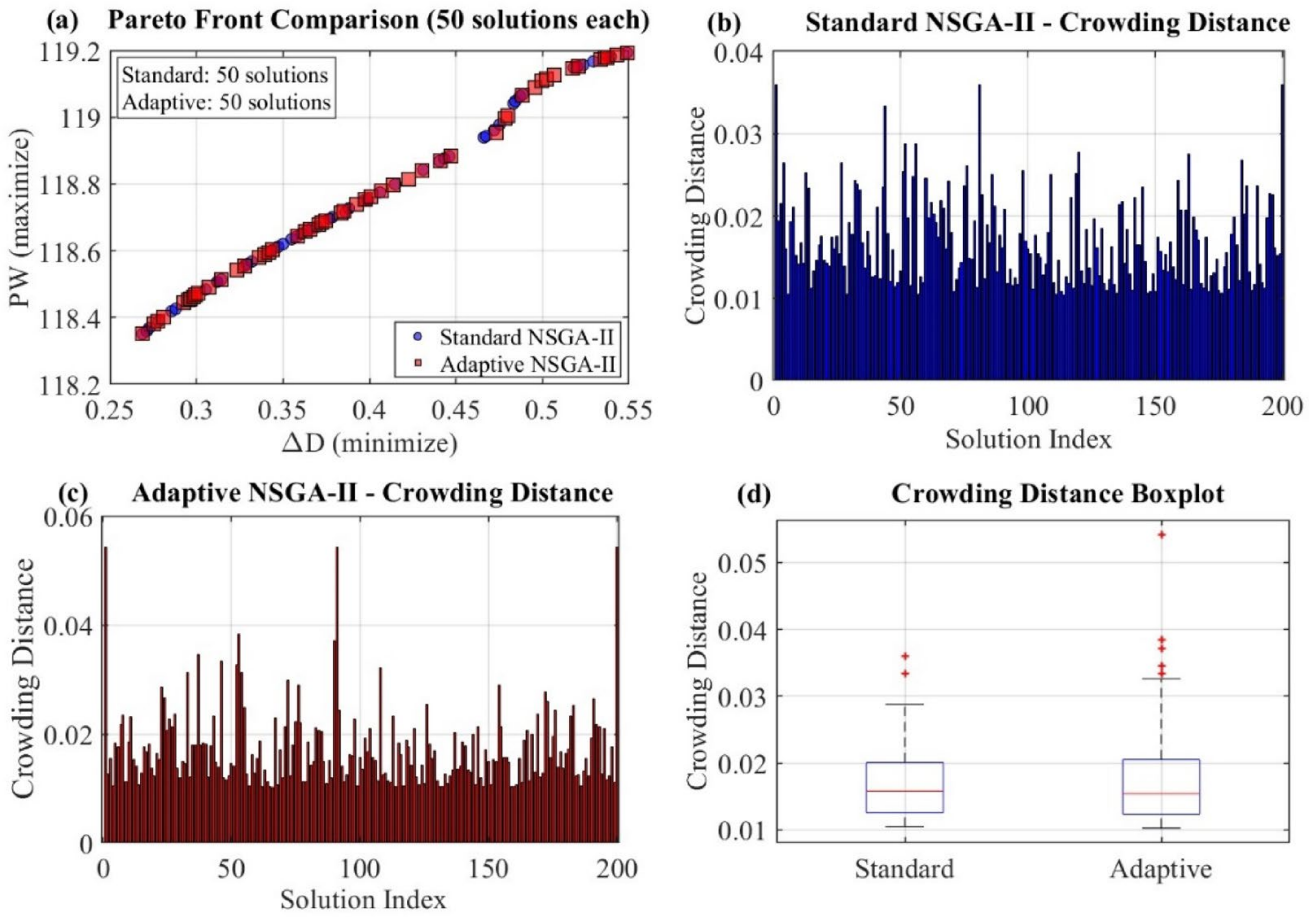
Pareto front and crowding distance comparison between standard NSGA-II and adaptive NSGA-II.

The Pareto-front distribution and diversity characteristics of the standard and adaptive NSGA-II algorithms are illustrated in Fig. 6. The Pareto fronts generated by both algorithms are presented in Fig. 6a. For visualization clarity, 50 representative solutions from each algorithm are plotted. The results reveal a clear trade-off relationship between *ΔD* and *PW*. As dimensional deviation decreases, the molded part weight tends to slightly decrease, indicating that achieving higher dimensional accuracy may reduce the level of melt packing and material retention in the cavity. Both algorithms generate Pareto fronts with nearly identical shapes, demonstrating their effectiveness in approximating the Pareto-optimal front. Nevertheless, the adaptive NSGA-II provides a slightly broader spread of solutions in certain regions of the front, offering more flexible alternatives for process parameter selection.

Solution diversity along the Pareto front was evaluated using the crowding distance metric. The crowding distance distributions for the standard and adaptive NSGA-II algorithms are shown in Fig. 6b and c, respectively. In both cases, the solutions are reasonably distributed along the Pareto front, indicating that population diversity is well preserved during the evolutionary process. The boxplot comparison in Fig. 6d shows that the adaptive NSGA-II tends to produce slightly larger crowding distance values on average, suggesting improved solution diversity. This improvement can be attributed to the adaptive mutation mechanism, which enhances exploration in the early search stage and helps avoid premature convergence.

To quantitatively evaluate the diversity characteristics of the obtained Pareto solutions, additional crowding-distance and hypervolume analyses were conducted for both the Standard and Adaptive NSGA-II algorithms, as summarized in Table 7. Compared with the Standard NSGA-II, the Adaptive NSGA-II exhibited slightly higher mean crowding-distance values, while the standard deviation, interquartile range, and maximum crowding distance increased by 35.0%, 23.2%, and 50.6%, respectively. These results indicate improved preservation of solution diversity and broader Pareto-front exploration capability. However, both approaches produced nearly identical hypervolume values, suggesting that the adaptive strategy primarily enhanced population diversity rather than convergence performance within the investigated objective space.

**Table 7:** Quantitative comparison of diversity and convergence metrics between Standard and Adaptive NSGA-II algorithms.

Metric	Standard NSGA-II	Adaptive NSGA-II	Relative Change (%)
Hypervolume (HV)	4.134	4.134	Approximately 0
Mean Crowding Distance	0.017	0.018	1.8
Standard Deviation	0.006	0.008	35
Maximum Crowding Distance	0.036	0.054	50.6
Interquartile Range (IQR)	0.007	0.009	23.2

Overall, both NSGA-II variants successfully generate well-distributed Pareto-optimal solutions representing the trade-off between dimensional deviation and molded part weight. Although the convergence behavior of the two algorithms is generally similar, the adaptive mutation strategy slightly improves solution diversity along the Pareto front, thereby providing a wider range of feasible process parameter combinations for practical decision-making.

### Decision-making using entropy-TOPSIS

After obtaining the Pareto-optimal solutions from NSGA-II, an entropy-weighted TOPSIS method was employed to identify the most suitable compromise solution. In this approach, the relative importance of the two objectives was determined objectively using the information entropy of the Pareto dataset, rather than being assigned subjectively.

As summarized in Table 8, the entropy values of *ΔD* and *PW* were 0.9651 and 0.9645, corresponding to diversification degrees of 0.0349 and 0.0355, respectively. The resulting normalized weights were 0.4954 for *ΔD* and 0.5046 for *PW*, indicating that both objectives contribute almost equally to the decision-making process, with *PW* having a slightly higher influence.

**Table 8:** Entropy-based weights of the optimization objectives.

Objective	Entropy (e_j_)	Diversification (d_j_)	Weight (w_j_)
*ΔD* (minimize)	0.9651	0.0349	0.4954
*PW* (maximize)	0.9645	0.0355	0.5046

$\:{e}_{j}$ denotes the entropy value, $\:{d}_{j}$ represents the diversification degree, and $\:{w}_{j}$ is the normalized weight calculated using the entropy weighting method.

**Fig. 7:**
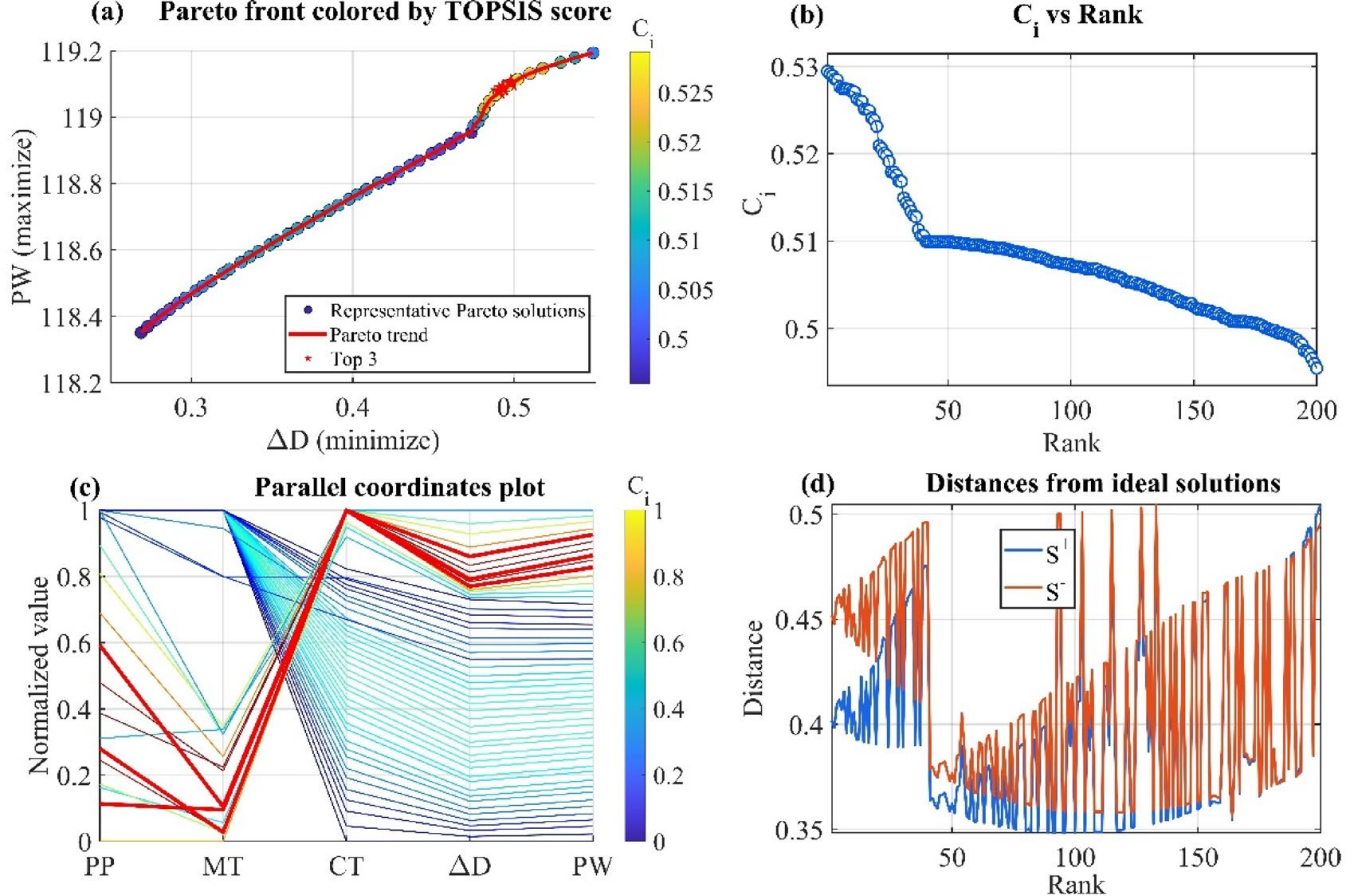
TOPSIS-based decision analysis of the Pareto solutions.

Based on these entropy-derived weights, the Pareto solutions were ranked using the TOPSIS closeness coefficient $\:{C}_{i}$. The ranking results are presented in Fig. 7. As shown in Fig. 7a, the Pareto front colored by the TOPSIS score reveals that the highest-ranked solutions are concentrated in the upper-right curved region of the front, where favorable trade-offs between *ΔD* and *PW* are achieved. These solutions correspond to the transition zone of the Pareto front, where further improvement of one objective would require a noticeable compromise in the other.

The variation of $\:{C}_{i}$ with respect to rank is illustrated in Fig. 7b. The closeness coefficient decreases smoothly from approximately 0.5295 to 0.495, indicating a stable and continuous ranking distribution. Figure 7c presents a parallel coordinates plot that highlights the distribution of the decision variables among the ranked solutions. The highest-ranked solutions are generally associated with relatively high packing pressure, moderate melt temperature, and the maximum cooling time, which jointly contribute to achieving lower *ΔD* and higher *PW*. The separation distances from the positive and negative ideal solutions, illustrated in Fig. 7d, further support the TOPSIS ranking results. Higher-ranked solutions are characterized by smaller $\:{S}^{+}$values and larger $\:{S}^{-}$values, which is consistent with the TOPSIS principle.

According to the entropy-weighted TOPSIS ranking, the best compromise solution corresponds to *PP* = 41.7 bar, *MT* = 225.5 °C, and *CT* = 60 s, yielding *ΔD* = 0.49 and *PW* = 119.09 with the highest closeness coefficient of $\:{C}_{i}=0.530$. The top-ranked Pareto solutions obtained from the entropy-weighted TOPSIS analysis are summarized in Table 9. The first three solutions exhibit very similar objective values and closeness coefficients, indicating that the optimal process parameter region is relatively stable.

**Table 9:** Top-ranked Pareto solutions obtained using entropy-weighted TOPSIS.

Rank	PP (bar)	MT (°C)	CT (s)	ΔD(mm)	PW (g)	(S^+^)	(S^−^)	(C_i_)
1	41.7	225.5	60.0	0.49	119.09	0.402	0.452	0.530
2	41.5	224.9	60.0	0.49	119.08	0.398	0.448	0.529
3	42.2	226.6	60.0	0.50	119.10	0.409	0.459	0.529

Note: $\:{S}^{+}$and $\:{S}^{-}$represent the distances from the positive and negative ideal solutions, respectively, while $\:{C}_{i}\:$denotes the TOPSIS closeness coefficient.

Overall, the entropy-weighted TOPSIS method provides an objective and effective framework for selecting the final process parameters from the Pareto set. By integrating entropy-based weighting with TOPSIS ranking, the approach reduces subjective bias and identifies a practically balanced solution that simultaneously satisfies the competing objectives.

### Experimental validation

To validate the effectiveness of the proposed NSGA-II–TOPSIS optimization framework, experimental verification was conducted using the final compromise solution selected by the entropy-weighted TOPSIS method together with two additional Pareto-optimal processing conditions. The selected processing parameters and corresponding validation results are summarized in Table 10.

**Table 10:** Experimental validation of the TOPSIS-selected solution and additional Pareto-optimal processing conditions.

Processing parameters			Results			
PP (bar)	MT (°C)	CT (s)	Response	Predicted	Experimental	Deviation (%)
41.7	225.5	60.0	*ΔD* (mm)	0.49	0.52	6.12
			*PW* (g)	119.09	117.07	1.70
45.0	240.0	49.0	*ΔD* (mm)	0.40	0.42	5.00
			*PW* (g)	118.77	117.26	1.27
45.0	240.0	30.0	*ΔD* (mm)	0.27	0.29	7.41
			*PW* (g)	118.35	117.13	1.03

The results show that the relative deviations between predicted and experimental values remained below 7.41% for *ΔD* and below 1.70% for *PW* across three validation cases. Although slightly larger deviations were observed for *ΔD* compared with *PW*, this behavior is considered reasonable because dimensional deviation is more sensitive to local shrinkage variation, residual stress relaxation, and geometric complexity during cooling and demolding. Overall, the validation results demonstrate the potential applicability of the proposed NSGA-II–TOPSIS optimization framework for identifying processing parameters that improve dimensional accuracy while maintaining adequate molded part weight across different regions of the Pareto front under industrial manufacturing conditions.

## Conclusion

This study proposed an integrated optimization and decision-making framework for improving the injection molding quality of a three-dimensional curved SGP. By combining BBD experimental design, RSM, NSGA-II optimization, and entropy-weighted TOPSIS decision analysis, the proposed framework enabled systematic identification of processing conditions that balance dimensional accuracy and molded part weight.

The statistical analysis showed that the developed quadratic RSM models provided satisfactory predictive capability within the investigated processing range. Among the investigated parameters, *PP* exhibited the strongest influence on both dimensional deviation and molded part weight, followed by *CT*, while *MT* showed a comparatively weaker effect. Both Standard and Adaptive NSGA-II algorithms successfully generated well-distributed Pareto-optimal solutions, with the Adaptive NSGA-II showing slightly improved diversity performance.

The entropy-weighted TOPSIS method identified an optimal parameter combination of *PP* = 41.7 bar, *MT* = 225.5 °C, and *CT* = 60 s. Additional experimental validation was performed not only for the TOPSIS-selected optimum solution but also for two supplementary Pareto-optimal processing conditions. The deviations between predicted and experimental responses remained below 7.41% for *ΔD* and below 1.70% for *PW* across all validation cases, indicating good agreement between the proposed framework and experimental observations. These results demonstrate the potential applicability of the proposed optimization framework for improving dimensional accuracy while maintaining adequate molded part weight under industrial manufacturing conditions.

Several limitations of the present study should be noted. First, dimensional deviation was evaluated using only three discrete inspection points, which may not fully represent the overall geometric deformation of the complex 3D curved part. Second, although the quadratic response surface model provided satisfactory prediction accuracy within the investigated range, it may have limited capability in capturing highly nonlinear process behavior. Finally, the post-Pareto decision-making process was based solely on entropy-weighted TOPSIS. Future studies may incorporate full-field geometric inspection methods, such as 3D scanning or coordinate measuring machine (CMM)-based surface analysis, to evaluate global deformation behavior more comprehensively. In addition, incorporating additional process parameters (e.g., injection speed, mold temperature, and holding time) together with advanced surrogate models (e.g., Gaussian Process Regression, Kriging, and ANN) could further improve prediction accuracy and extend the applicability of the proposed framework. Finally, comparing different MCDM approaches such as VlseKriterijumska Optimizacija I Kompromisno Resenje (VIKOR) and the Analytic Hierarchy Process (AHP), with entropy-weighted TOPSIS may provide further insight into the robustness of the selected compromise solution.

## Data Availability

The datasets generated and analysed during the current study are available from the corresponding author on reasonable request.

## Code availability

The custom MATLAB code used for response surface model fitting, standard and adaptive NSGA-II optimization, Pareto-front analysis, hypervolume and crowding-distance evaluation, and entropy-weighted TOPSIS ranking has been deposited in Zenodo and is publicly available at: 10.5281/zenodo.20673954.

## References

[1] Singh, G. & Verma, A. A Brief Review on injection moulding manufacturing process. *Mater. Today Proc.* **4**, 1423–1433 (2017).

[2] Chen, Z. & Turng, L. S. A review of current developments in process and quality control for injection molding. *Adv. Polym. Technol.* **24**, 165–182 (2005).

[3] Zhao, M. & Tang, Z. Comprehensive analysis of the injection mold process for complex fiberglass reinforced plastics with conformal cooling channels using multiple optimization method models. *Processes* **13**, 2803 (2025).

[4] Zhao, N.-y, Lian, J.-y, Wang, P.-f & Xu, Z.-b. Recent progress in minimizing the warpage and shrinkage deformations by the optimization of process parameters in plastic injection molding: A review. *Int. J. Adv. Manuf. Technol.* **120**, 85–101 (2022). 35194289 10.1007/s00170-022-08859-0 PMC8831005

[5] Kurtaran, H. & Erzurumlu, T. Efficient warpage optimization of thin shell plastic parts using response surface methodology and genetic algorithm. *Int. J. Adv. Manuf. Technol.* **27**, 468–472 (2006).

[6] Kameda, T., Takahashi, T. & Koyama, K. Effect of packing pressure on molding shrinkage distribution of injection molded parts of acrylonitrile-styrene (AS) resin. *Seikei-Kakou* **17**, 571–579 (2005).

[7] Wang, Q. et al. Effect of process parameters on repeatability precision of weight for microinjection molding products. *Adv. Polym. Technol.* **2019**, 2604878 (2019).

[8] Annicchiarico, D. & Alcock, J. R. Review of factors that affect shrinkage of molded part in injection molding. *Mater. Manuf. Process.* **29**, 662–682 (2014).

[9] Feng, Q., Liu, L. & Zhou, X. Automated multi-objective optimization for thin-walled plastic products using Taguchi, ANOVA, and hybrid ANN-MOGA. *Int. J. Adv. Manuf. Technol.* **106**, 559–575 (2020).

[10] Li, S. et al. Optimization of injection molding process of transparent complex multi-cavity parts based on Kriging model and various optimization techniques. *Arab. J. Sci. Eng.* **46**, 11835–11845 (2021).

[11] Tran, C. C., Nguyen, T. T. & Nguyen, V. T. Multi-objective optimisation in turning AISI 304 stainless steel: an integration of the Taguchi method, response surface methodology, and NSGA-II. *International Journal of Industrial and Systems Engineering* **50**, 413–432 (2025).

[12] Tran, C. C. Modelling and optimization of surface roughness and material removal rate in milling SKD11 Using GMDH and NSGA-II. *Int. J. Mech. Eng. Robot Res.* **13**, 618–627 (2024).

[13] Verma, S., Pant, M. & Snasel, V. A comprehensive review on NSGA-II for multi-objective combinatorial optimization problems. *IEEE access* **9**, 57757–57791 (2021).

[14] Tran, C. C. & Nguyen, V. T. Optimization of Filling Time and Volumetric Shrinkage Rate in Simulation of Plastic Product Injection Molding Process Using RSM and NSGA-II. *Jordan J. Mech. Ind. Eng.* **19**, 67–77 (2025).

[15] Li, S., Fan, X., Huang, H. & Cao, Y. Multi-objective optimization of injection molding parameters, based on the Gkriging-NSGA-vague method. *J. Appl. Polym. Sci.* **137**, 48659 (2020).

[16] Jung, J., Park, K., Lee, H., Cho, B. & Ryu, S. Comparative study of multi-objective Bayesian optimization and NSGA‐III based approaches for injection molding process. *Advanced Theory and Simulations*10.1002/adts.202400135 (2024).

[17] Godec, D., Panđa, F., Tujmer, M. & Monkova, K. Molded part warpage optimization using inverse contouring method. *Polymers* **17**, 2278 (2025). 40942195 10.3390/polym17172278 PMC12431507

[18] Solanki, B. S., Singh, H. & Sheorey, T. Effect of injection molding cycle time on shrinkage and weight of manufactured polymer gear. *Materials Today: Proceedings* **62**, A1–A6 (2022).

[19] Moayyedian, M., Qazani, M. R. C., Amirkhizi, P. J., Asadi, H. & Hedayati-Dezfooli, M. Multiple objectives optimization of injection-moulding process for dashboard using soft computing and particle swarm optimization. *ASEE North. Cent. Sect. Conf.* **14**, 23767 (2024). 10.1038/s41598-024-62618-7 PMC11467200 39389993

[20] Roy, B., Krug, S. & Hutschenreuther, T. Real-Time Optimal Parameter Recommendation for Injection Molding Machines Using AI with Limited Dataset. *AI* **7**, 49 (2026).

[21] Zhou, H., Zhang, S. & Wang, Z. Multi-objective optimization of process parameters in plastic injection molding using a differential sensitivity fusion method. *Int. J. Adv. Manuf. Technol.* **114**, 423–449 (2021).

[22] Kariminejad, M. et al. Single and multi-objective real-time optimisation of an industrial injection moulding process via a Bayesian adaptive design of experiment approach. *Sci. Rep.* **14**, 29799 (2024). 39616254 10.1038/s41598-024-80405-2 PMC11608349

[23] Liu, F., Pang, J. & Xu, Z. Multi-objective optimization of injection molding process parameters for moderately thick plane lens based on PSO-BPNN, OMOPSO, and TOPSIS. *Processes* **12**, 36 (2023).

[24] Liu, X., Fan, X., Guo, Y., Cao, Y. & Li, C. Multi-objective optimization of GFRP injection molding process parameters, using GA-ELM, MOFA, and GRA-TOPSIS. *Transactions of the Canadian Society for Mechanical Engineering* **46**(1), 37–49 (2022).

[25] Deb, K., Pratap, A., Agarwal, S. & Meyarivan, T. A fast and elitist multiobjective genetic algorithm: NSGA-II. *IEEE Transactions on Evolutionary Computation* **6**, 182–197 (2002).

[26] Behzadian, M., Otaghsara, S. K., Yazdani, M. & Ignatius, J. A state-of the-art survey of TOPSIS applications. *Expert Systems with Applications* **39**, 13051–13069 (2012).

[27] Guevara-Morales, A. & Figueroa-López, U. Residual stresses in injection molded products. *Journal of materials science* **49**, 4399–4415 (2014).

[28] Switzner, N. T., Abubakr, D. O., AbdulAziz, N. N., Rasheed, F. F. & Jamal, L. H. Effects of injection molding parameters and cooling conditions on the dimensional stability and mechanical properties of polypropylene boxes for automotive batteries. *Discov. Mech. Eng.* **4**, 63 (2025).

[29] Tsai, H. H., Wu, S. J., Liu, J. W., Chen, S. H. & Lin, J. J. Filling-balance-oriented parameters for multi-cavity molds in polyvinyl chloride injection molding. *Polymers* **14**, 3483 (2022). 36080557 10.3390/polym14173483 PMC9459958

[30] Kamal, M., Lai-Fook, R. & Hernandez‐Aguilar, J. Residual thermal stresses in injection moldings of thermoplastics: a theoretical and experimental study. *Polymer Engineering & Science* **42**, 1098–1114 (2002).

[31] Mokhtar, W. & Nasir, S. B. *in ASEE North Cent. Sect. Conf* Vol. 44634 (American Society for Engineering Education, 2024).

